# Neural correlates of heterotopic facilitation induced after high frequency electrical stimulation of nociceptive pathways

**DOI:** 10.1186/1744-8069-7-28

**Published:** 2011-04-20

**Authors:** Emanuel N van den Broeke, Casper H van Heck, Clementina M van Rijn, Oliver HG Wilder-Smith

**Affiliations:** 1 Department of Anesthesiology, Pain & Palliative Medicine, Pain & Nociception Neuroscience Research Group, Radboud University Nijmegen Medical Centre, P.O. Box 9101, 6500 HB Nijmegen, The Netherlands; 2 Donders Institute for Brain, Cognition and Behavior, Radboud University Nijmegen, P.O. Box 9104, 6500 HE Nijmegen, The Netherlands

## Abstract

**Background:**

High frequency electrical stimulation (HFS) of primary nociceptive afferents in humans induce a heightened sensitivity in the surrounding non-stimulated skin area. Several studies suggest that this heterotopic effect is the result of central (spinal) plasticity. The aim of this study is to investigate HFS-induced central plasticity of sensory processing at the level of the brain using the electroencephalogram (EEG). To this end we measured evoked potentials in response to noxious electrical pinprick-like stimuli applied in the heterotopic skin area before, directly after and 30 minutes after HFS.

**Results:**

We observed potential cortical electrophysiological correlates of heterotopic facilitation. Two different cortical correlates were found; the first one was a lateralized effect, i.e. a larger N100 amplitude on the conditioned arm than the control arm 30 minutes after end of HFS. This was comparable with the observed lateralized effect of visual analogue scale (VAS) scores as response to the mechanical punctate stimuli. The second correlate seems to be a more general (non-lateralized) effect, because the result affects both arms. On average for both arms the P200 amplitude increased significantly 30 minutes after end of HFS with respect to baseline.

**Conclusions:**

We suggest that for studying heterotopic nociceptive facilitation the evoked brain response is suitable and relevant for investigating plasticity at the level of the brain and is perhaps a more sensitive and reliable marker than the perceived pain intensity (e.g. VAS).

## 1. Background

Long-term potentiation (LTP) is a cellular model for synaptic plasticity [1] and reflects increase of synaptic strength [2]. It has been shown by both in vivo (anesthesized animals) and in vitro (slice preparations) studies that LTP can also be induced in the nociceptive system in response to high frequency stimulation (HFS) [3-5]. It is believed that LTP in nociceptive pathways may underlie some forms of hyperalgesia [6,7].

According to Klein et al. [8] nociceptive LTP can also be elicited in humans after high frequency electrical stimulation (HFS) of primary nociceptive afferents. They demonstrated the effectiveness of HFS in inducing LTP by observing potential perceptual correlates, e.g. increased subjective pain perception in response to single electrical stimuli. In this context, LTP is manifested as a heightened sensitivity in the stimulated area (homotopic effect). Besides this homotopic effect, Klein et al. also observed an increased subjective pain perception in the area surrounding the stimulated area (heterotopic effect). This heterotopic effect was observed in response to mechanical punctate stimuli [8]. Mechanical punctate stimuli evokes a sharp pain sensation which is believed to be signaled by myelinated Aδ nociceptors [9,10]. Several studies suggest that this heterotopic effect, which is also observed in other pain inducing models, is the result of central (spinal) plasticity [11-14].

The aim of this study is to investigate HFS-induced central plasticity of sensory processing at the level of the brain using the electroencephalogram (EEG). To this end we measured evoked potentials [15] to noxious electrical pinprick-like stimuli applied in the heterotopic skin area before, directly after and 30 minutes after HFS. Our hypothesis was that facilitation of central nociceptive processing would be visible as differences in amplitude of the evoked potentials for the stimulated vs. control condition.

## 2. Materials and Methods

### 2.1 Participants

Eighteen healthy woman volunteers (mean age 24 yr; range, 20 - 31 yr) participated in the experiment. Subjects were excluded from the study if they had a history of psychiatric or neurological disorder, used medication, or suffered from pre-existing pain or pain syndromes. All participants signed an informed consent form. Approval for the experiment was obtained from the local Ethical Committee.

### 2.2 Design

#### 2.2.1 Experimental conditioning: high frequency electrical stimulation (HFS)

**Figure 1:**
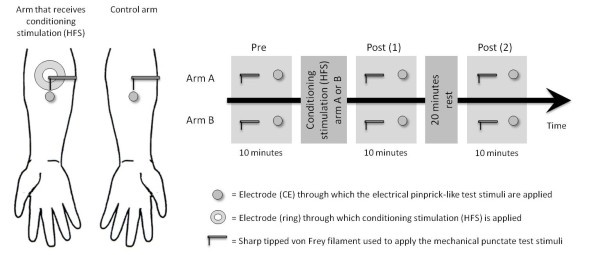
**Experimental set-up and design**. *Left: *Positions of the ring electrode used for experimental conditioning and the concentric electrode (CE) used to apply the electrical pinprick-like test stimuli. The conditioning electrode was placed 5 cm and the concentric electrode 7 cm from the cubita fossa. The mechanical punctate test stimuli were delivered between 6 and 7 cm from the cubita fossa on the distal axis. *Right: *Time-table of the experiment.

Subjects received trains of 100 Hz (pulse width; 2 ms) for 1 sec. repeated 5 times at 10 sec intervals with an intensity of 20 × detection threshold on the forearm 5 cm distal to the fossa cubita. The stimulation trains were delivered via a ring electrode (figure 1) consisting of 16 blunt stainless steel pins with a diameter of 0.2 mm protruding 1 mm from the base. The 16 pins are placed in a circle with a diameter of 10 mm and serve as cathode. A stainless steel reference electrode which serves as anode is concentrically located and has an inner diameter of 22 mm and an outer diameter of 40 mm. This electrode is specially designed to activate superficial nociceptive afferents with less concomitant recruitment of tactile afferents [8]. The opposite arm to the one receiving conditioning stimulation served as control. In order to avoid interference of lateral dominance, the stimulated arm (HFS) was balanced (dominant or not dominant) across subjects.

#### 2.2.2 Variables measured

#### 2.2.2.1 Behavioral measurements (perceptual correlates of heterotopic facilitation)

##### 2.2.2.1.1 High frequency electrical stimulation

Changes in pain perception during experimental conditioning stimulation (HFS) were tested by asking the subjects after each train to rate the amount of pain on a Visual Analogue Scale (VAS) ranging from 0 cm = ''no pain" to 10 cm = ''unbearable pain".

##### 2.2.2.1.2 Electrical pinprick-like test stimuli

In order to quantify the heterotopic effects as a result of experimental conditioning stimulation, blocks of twenty single noxious pulses (monopolar square wave; duration 0.5 ms) were applied on both arms (conditioned and control) before, directly after and 30 minutes after the experimental conditioning. We chose thirty minutes as a late measurement after conditioning stimulation because Klein et al. [16] showed that punctate hyperalgesia develops immediately after HFS and then increases slightly over the next 40 min, peaking between 40 and 60 min after HFS. Thus we chose thirty minutes after HFS in order to be sure the effect was well-established without being in the declining phase.

For the conditioned arm, the stimuli were applied at 2.5 cm outside the area of conditioning stimulation, on the control arm the same area was used. The pulses were delivered with a random inter-pair interval ranging from 7 to 10 seconds via a concentric electrode (CE). Because of its concentric design and small anode-cathode distance this stimulating electrode produces a high current density at relatively low current intensities [17]. In this way depolarization is preferentially limited to nociceptive Aδ fibers in the superficial layer of the dermis without recruitment of deeper lying non-nociceptive Aβ fibers. Stimulation with this electrode produces a clear and well localized pinprick-like painful sensation [17,18]. In order to quantify the amount of pain as a result of this pinprick-like stimulation, subjects were asked to rate, at random times within a train of 5 single pulses, the pain intensity of the last received stimulus on a VAS. The VAS ranged from 0 cm = ''no pain" to 10 cm = ''unbearable pain" and was used by the subject by moving the mouse pointer (vertical line) on a horizontal bar.

The single pulses were delivered through the CE using a constant current stimulator (Digitimer DS7A, Digitimer UK) and with an intensity of 150% of the individual pinprick pain threshold. This individual pain threshold was determined by an ascending sequence of increased current intensities (single square wave current pulse; duration 5 ms) starting from 0 mA and with steps of 0.1 mA. This procedure stopped when the pain threshold (pricking painful sensation) was achieved, as verbally reported by the subjects. This threshold determination protocol was performed twice and the mean was used in the experiment.

During stimulation, subjects were comfortably seated in a chair and were instructed to passively perceive the stimuli with eyes closed, without making any movements. A computer display was placed in front of the subject (0.5 m) together with a computer mouse. The display was used to display the VAS, preceded by a tone (65 dB). Participants were instructed to open their eyes after the tone and use the mouse to mark the VAS, after which they closed their eyes again.

##### 2.2.2.1.3 Mechanical punctate test stimuli

A second behavioral test was used to test for effects of HFS on mechanical punctate stimuli. A calibrated sharp-tipped von Frey monofilament (size: 6.1, target force: 980 mN, Sammons Preston Rolyan, USA) was pressed on the heterotopic skin area (in between the CE and the area of conditioning stimulation) of the conditioned arm and on the same area of the control arm. After each stimulus, subjects were asked to rate the sensation on a modified VAS ranging from 0 cm = ''I feel nothing" to 10 cm = ''unbearable pain" and at 5 cm a vertical line representing the transition from non-painful to painfulness. The VAS was marked by drawing a vertical line on a horizontal bar.

#### 2.2.2.2 Electrophysiological measurements (ERP correlates of heterotopic facilitation)

##### Evoked potential waveforms

In order to measure the pinprick-like evoked brain responses, a multi-channel (32 channels) EEG (Brainvision system) was recorded (band-pass 0.1-100 Hz, sample frequency 500 Hz) during the electrical pinprick-like stimulation. The electrodes were mounted in an elastic electrode-cap and arranged according to the international 10-20 system. Electrode CPz was used as reference. Eye movements were detected by horizontal and vertical electrooculogram (EOG) recordings. Horizontal EOG was measured from the outer canthus of the left eye, and vertical EOG supra orbitally to the left eye. Impedance was kept under the 20 kΩ for all leads.

### 2.3 Procedure (figure 1)

At the beginning of the experiment individual pinprick-like pain thresholds for the single electric pulse stimulation were determined. The arm on which this pain threshold was determined (conditioned or control arm) was balanced across subjects. After this pain threshold determination subjects received two blocks (one at each arm) of electrical pinprick-like test stimuli as well as a single mechanical punctate stimulus (pre measurement). The sequence applied was balanced across subjects; one half of the subjects received first the electrical and then the mechanical stimulus, the reversed sequence was received by the other half of the subjects. The same procedure was applied regarding which arm was tested first. After the baseline measurement (pre) the experimental conditioning (HFS) followed. After receiving conditioning stimulation two post measurements (post (1) and post (2)) followed. Post (1) was directly after conditioning stimulation and post (2) 30 minutes after. The procedures for these two post measurements were the same as for the pre measurement.

### 2.4 Signal analysis

Evoked potential waveforms measured at the vertex (Cz electrode) were extracted from the EEG off-line with Brain Vision Analyzer software version 1.05. As a first step the continuous EEG was down-sampled to 500 Hertz (Hz) and high-pass filtered at 1 Hz and low-pass filtered at 30 Hz. After that the EEG was segmented, based on the onset of the stimulus, into epochs from -100 ms pre-stimulus to 500 ms post-stimulus with a total period of 600 ms. Bad segments containing ocular artifacts were removed using the Gratton-Coles method [19]. Segments were also inspected for other artifacts like muscle or jaw and line noise activity and were removed if necessary. As a last step baseline correction (-100 - 0 ms) was applied to all segments which were than averaged to get a subject-specific evoked potential waveform. For computing grand average waveforms for each condition separately all subject-specific average waveforms were averaged.

Based on morphology and latency of the grand average waveform three distinct amplitudes were defined: (1) N100; most negative deflection after stimulus onset, (2) P200; first positive deflection that follows the N100 and (3) P300; most positive deflection after P200. To quantify possible differences in the grand average evoked potential waveform between different conditions the mean amplitude within a specified time window (based on the grand average waveforms) is calculated in each subject-specific average [20]. The time window of the N100 is 70-120 ms; P200 is 180-220 ms and P300 is 260-310 ms. The rationale for using the mean activity instead of the more commonly used maximal peak value is that the fewer trials included in the subject-specific average, the more residual noise is superimposed on the maximal peak. As a result the maximal peak of the subject-specific average will be determined by residual noise rather than by the peak of interest. Therefore the mean amplitude is calculated instead of the maximal peak because the first one is more stable.

### 2.5 Statistical analysis

For statistical analysis the software SPSS v. 16.0 was used. A General Linear Model (GLM) repeated measures ANOVA analysis was used to test whether there are statistically significant differences regarding the behavioral and electrophysiological measurements with respect to the *time of measurement *(pre, post1 and post2) and *place *(control and conditioned arm). In all tests the significance level was set at *p *< .05.

## 3. Results

### 3.1 Behavioral measurements

#### 3.1.1 High frequency electrical stimulation

**Figure 2:**
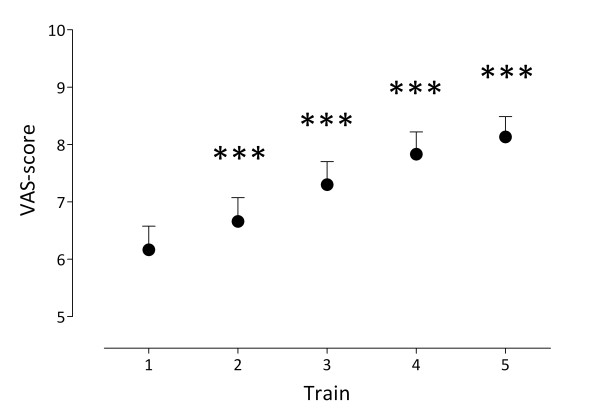
**Mean (and SEM) VAS-scores as response to the conditioning high frequency stimulation**. A repetition of trains of high frequency electrical stimuli resulted in a gradual increase of pain perception *** = *p *< .001.

The GLM repeated measures ANOVA analysis revealed a significant main effect of *Time *(F (4,14) = 17.481, *p *< .001, eta^2 ^= .833). Univariate within-subject contrasts showed that repetition of trains of high frequency electrical stimuli resulted in a gradual increase of pain perception (figure 2):

- train 1 (M = 6.2) vs. train 2 (M = 6.7) : F (1,17) = 45.911, *p *< .001, eta^2 ^= .730

- train 2 vs. train 3 (M = 7.3) : F (1,17) = 19.508, *p *< .001, eta^2 ^= .534

- train 3 vs. train 4 (M = 7.8) : F (1,17) = 42.667, *p *< .001, eta^2 ^= .715 and

- train 4 vs. train 5 (M = 8.1) : F (1,17) = 20.864, *p *< .001, eta^2 ^= .551.

#### 3.1.2 Mechanical punctate test stimuli

Regarding the mechanical test stimuli the GLM repeated measures ANOVA analysis revealed a significant main effect of *Time *(F (2,16) = 4.161, *p *= .035, eta^2 ^= .342). The univariate within-subject contrasts revealed that on average over both arms the VAS-score increased significantly between pre (M = 1.9) and post (1) (M = 2.5) (F (1,17) = 8.801, *p *= .009, eta^2 ^= .341) and between pre and post (2) (M = 2.6) (F (1,17) = 4.753, *p *= .044, eta^2 ^= .218. There is also a significant main effect of *Arm *(F (1,17) = 7.694, *p *= .013, eta^2 ^= .312). On average over the three time points the VAS-score is different between the two arms; conditioned arm M = 2.6 and control arm M = 2.1.

**Figure 3:**
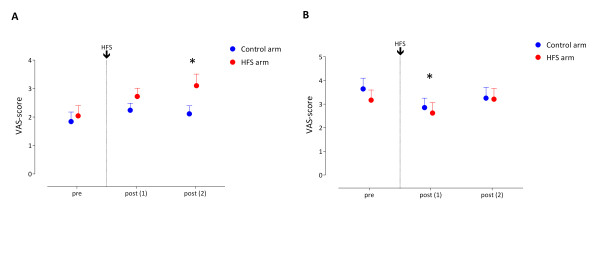
**Behavioral measurements**. **A)** Mean (and SEM) VAS-scores as response to mechanical punctate test stimulation. The VAS-score observed at the conditioned arm was significantly higher than the VAS-score observed at the control arm 30 minutes after experimental conditioning stimulation (post (2)) * = *p *< .05. **B) **Mean (and SEM) VAS-scores as response to electrical pinprick-like test stimulation. Averaged for both arms a significant decrease of the VAS-score was present between baseline (pre) and post (1) experimental conditioning stimulation * = *p *< .05.

More interestingly a significant *Time × Arm *interaction effect (F (2,16) = 3.952, *p *= .040, eta^2 ^= .331) was found. The univariate within-subject contrasts showed a statistically significant difference in VAS-score on post2 (versus pre) between the two arms (conditioned vs. control arm) (F (1,17) = 8.331, *p *= .010, eta^2 ^= .329). The VAS-score observed at the conditioned arm was significantly higher (M = 3.1) than the VAS-score observed at the control arm (M = 2.1) 30 minutes after experimental conditioning stimulation (figure 3A).

Post hoc tests (paired t-tests) showed a significant increase in VAS-score of the conditioned arm between pre (M = 2.0) and post (1) (M = 2.7) (t (17) = -2,854, *p *= .011) and between pre (M = 2.0) and post (2) (M = 3.1) (t (17) = -2,892, *p *= .010). *P*-values are corrected for multiple comparisons using the Bonferroni correction. No significant changes in VAS-scores were observed for the control arm.

#### 3.1.3 Electrical pinprick-like test stimuli

For the VAS-score observed during the electrical pinprick-like stimulation the GLM repeated measures ANOVA analysis revealed a significant main effect of *Time *(F (2,16) = 6.218, *p *= .010, eta^2 ^= .437). The univariate within-subject contrasts revealed that on average over both arms a significant decrease was present of the VAS-score between pre (M = 3.4) and post1 (M = 2.7) experimental conditioning stimulation (F (1,17) = 12.852, *p *= .002, eta^2 ^= .431) (figure 3B).

### 3.2 Electrophysiological measurements

#### 3.2.1 Evoked potential waveforms

**Figure 4:**
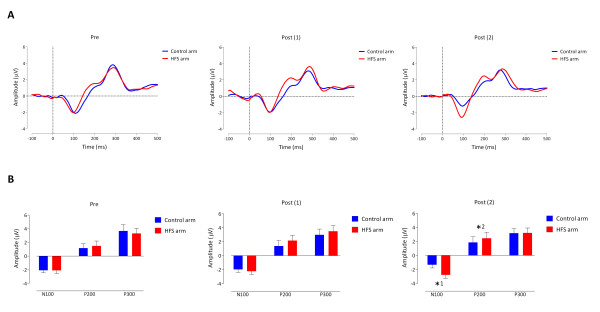
**Electrophysiological measurements**. **A) **Grand average evoked potential waveforms. Plotted are the grand averaged evoked potentials waveforms for each measurement (pre, post (1) and post (2)) compared between the two arms (control vs. conditioned). Dotted line on X-axis represents stimulus onset. Upward is positive and downward is negative charge. **B) **Histograms representing the N100, P200 and P300 evoked potential amplitudes for each arm (control vs. conditioned) at every measurement (pre, post (1), post (2)). *^1 ^The N100 amplitude observed at the conditioned arm was significantly (*p *< .05) larger than the N100 amplitude observed at the control arm 30 minutes after experimental conditioning stimulation (post (2)). *^2 ^Averaged for both arms the P200 amplitude significantly (*p *< .05) increased between baseline (pre) and 30 minutes after experimental conditioning stimulation (post (2)).

The grand average evoked potential waveforms for each measurement (pre, post(1) and post (2)) and arm (conditioned vs. control) as well as the means (and SEM) of the distinct N100, P200 and P300 amplitudes are summarized in figure 4A and 4B.

#### 3.2.2 N100 amplitude

The GLM repeated measures ANOVA analysis revealed a significant *Time × Arm *interaction effect for the N100 amplitude (F (2,16) = 4.891, *p *= .022, eta^2 ^= .379). The univariate within-subject contrasts showed a statistically significant difference in N100 amplitude between the two arms (conditioned vs. control arm) at post (2) (F (1,17) = 6.116, *p *= .024, eta^2 ^= .265). The N100 amplitude observed at the conditioned arm (M = -2.4) was significantly larger than the N100 amplitude observed at the control arm (M = -1.1) 30 minutes after experimental conditioning stimulation (figure 4B).

#### 3.2.3 P200 amplitude

For the P200 amplitude the GLM repeated measures ANOVA analysis revealed a significant main effect of *Time *(F (2,16) = 4.595, *p *= .027, eta^2 ^= .365). The univariate within-subject contrasts revealed on average for both arms a significant increase of the P200 amplitude between pre (M = 1.4) and post (2) (M = 2.2) experimental conditioning stimulation (F (1,17) = 8.215, *p *= .011, eta^2 ^= .326) (figure 4B).

#### 3.2.4 P300 amplitude

No significant differences were found on the P300 amplitude (figure 4B).

## 4. Conclusion and Discussion

This study has shown that conditioning HFS resulted in significant heterotopic effects after stimulation. These heterotopic effects included:

(1) an enhanced perceived intensity in response to mechanical punctate stimulation observed at the conditioned arm (in comparison with control arm) 30 minutes after HFS;

(2) averaged for both arms we observed a decreased perceived intensity (VAS) as response to electrical pinprick-like stimulation (in comparison with baseline) directly after HFS;

(3) an enhanced evoked brain response around 100 ms (N100) observed at the conditioned arm (in comparison with control arm) 30 minutes after HFS;

(4) averaged for both arms we observed an enhanced evoked brain response around 200 ms (P200) (in comparison with baseline) 30 minutes after HFS and

(5) no effects on P300 amplitude.

### 4.1. Perceptual correlate of heterotopic facilitation 30 minutes after HFS

The observed increased perceived intensity to heterotopically applied mechanical punctate stimuli after HFS is in agreement with Klein et al. [8] and Van Den Broeke et al. [21] and likely involves heterosynaptic facilitation [11,13]. At present it is still unclear which underlying mechanism(s) is/are responsible for the development of this heterosynaptic effect [11,12].

There are studies reporting evidence for a role of mechanosensitive myelinated Aδ nociceptors in mediating the sharp pain evoked by mechanical punctate stimuli in healthy skin and in the heterotopic skin site after the application of capsaicin [9,10]. In the present study, an increase in VAS-score to sharp tipped von Frey monofilament stimulation was indeed observed after HFS, but the ratings of the mechanical stimuli at baseline (before conditioning) were not rated as being painful. Thus, strictly speaking, we did not demonstrate hyperalgesia, for which the stimulus should be rated as painful before conditioning stimulation and should increase in rating after the intervention [23]. The observed effect cannot be labeled as allodynia either, because then the non-noxious stimulus should become painful [23] after HFS but this is clearly not the case, either. Two factors may play a role in this difference between our results and those of other groups studying human HFS. Firstly, the other groups [8] did not use a scale allowing intensity rating in the non-painful range - thus making it possible that they missed the non-painful nature of their stimuli. Secondly, there may have been differences in the nature of punctate stimulation used between the groups.

The two stimuli (mechanical, electrical) used in this study for perceptual correlates of heterotopic facilitation are in principle differently processed by the peripheral nervous system. Electrical stimuli directly depolarize the afferent nerve fiber, bypassing the processes related to receptor transduction. They therefore allow better synchronization of the afferent input. In contrast, mechanical stimuli are processed via mechanoreceptor transduction at the nerve ending. Theoretically, the mechanical stimulus is selective in activating mechanosensitive fibers, while electrical stimuli are not. Moreover, mechanical stimulation is a natural stimulus but electrical is not.

### 4.2 Electrophysiological correlate of heterotopic facilitation 30 minutes after HFS

In contrast to the mechanical stimuli, the electrical noxious pinprick-like stimuli were rated as painful because at the beginning of the experiment we determined the individual pain threshold as a basis for subsequent suprathreshold stimulation during the experiment. We observed an enhanced N100 amplitude (in comparison with control arm) evoked by these stimuli to be present 30 minutes after HFS. This agrees with the observed enhanced VAS-score (in comparison with the control arm) to the heterotopically applied mechanical stimuli, suggesting that the N100 amplitude might be a electrophysiological correlate of heterotopic facilitation. From a neurophysiological point of view the amplitude of the evoked potential waveform represents the synchronized activity of the underlying neural population [24]. Thus a larger EP amplitude means that more (cortical) neurons fire synchronously. Because the N100 is a relatively early component it probably reflects an early stage of sensory processing. It is interesting to note that we did not observe a similar effect as found on the N100 amplitude in the VAS scores on the measurement 30 minutes after HFS. Clearly the facilitated electric input was not subjectively detectable for the subjects for these stimuli.

Besides the enhanced N100 we also observed an enhanced P200 amplitude (in comparison with baseline and averaged for both arms) on the post (2) measurement. At present it is still unclear what process the P200 amplitude reflects but because the effect involved both arms it suggests a more general (non-lateralized) effect.

An interesting question is whether these observed changes in ERP amplitudes are solely the result of spinal changes or also supra spinal or (sub) cortical changes. Based on animal studies, it has been suggested that LTP in cortical structures, e.g. anterior cingulated cortex (ACC), might also accompany peripheral nociceptive input [25]. However, while the ERP effects seen in our study must originate in the brain (cortex), it is evident that the present study does not permit definitive distinction as to the origin of the changes observed. Furthermore, polysynaptic evoked responses, such as the ERP, are not suitable for directly studying variations in monosynaptic strength such as caused by LTP [6].

To date there is but one animal (PET) study localizing changes in the metabolic response to peripheral nociceptive input after HFS of the sciatic nerve to the brain [26]. In this study the authors observed an increase in metabolic response (in comparison to sham) directly after HFS in the primary somatosensory cortex congruent with the area of the stimulated limb. However, this effect was already fading 150 min after HFS. Interestingly, the authors did observe an increase in brain activity (in comparison to sham) on the measurement 150 minutes after HFS in areas such as: amygdala (including adjacent cortical areas and striatum), periaquaductal grey (PAG) and rostral ventromedial medulla (RVM) [26].

### 4.3. Decrease of pain perception directly after HFS

Directly after HFS we observed a decrease in perceived pain intensity in response to the electrical pinprick-like stimuli. Remarkably, we could not detect a correlate of this observed VAS effect in the evoked waveforms in the EEG. Several hypotheses could be put forward to explain this decrease in perceived pain intensity. One possibility is to ascribe the effect to habituation; a decrease in response to a stimulus when that stimulus is presented repeatedly [27]. Alternatively, the observed VAS effect could be similar to the effect observed after heterotopic noxious conditioning stimulation (HNCS) paradigms [28]. In this paradigm the pain intensity to a 'test' stimulus before and after a 'conditioning' stimulus (e.g. Ice water bath) is measured. The conditioning stimulus is typically applied to another body part than that of the test stimulus. The observed effect is usually a reduction of the test pain intensity after the conditioning stimulus, an effect attributed to diffuse noxious inhibitory controls (DNIC), demonstrated in both animals [29] and humans [28]. It is believed that DNIC and HNCS are manifestations of the involvement of the descending neural endogenous analgesia system [28,29]. A final possible explanation is the comparison effect. Here, the test stimuli applied directly after HFS could be compared by the subject with the conditioning HFS. After HFS, test stimuli might then be judged to be less painful.

### 4.4. Potential implications

To our knowledge this is the first study that investigated the heterotopic effect induced after HFS in humans with evoked potentials as response to noxious electrical pinprick-like stimuli. We observed potential cortical electrophysiological correlates of heterotopic facilitation.

Two different cortical correlates were found; the first one was a lateralized effect, i.e. a larger N100 amplitude on the conditioned arm than the control arm 30 minutes after end of HFS. The second correlate seems to be a more general (non-lateralized) effect, because the result affects both arms. On average for both arms the P200 amplitude increased significantly 30 minutes after end of HFS with respect to baseline. It is interesting to note that both effects appears only after 30 minutes after HFS which was remarkably similar for the observed lateralized effect of VAS-scores as response to the mechanical punctate stimuli. This in contrast with the VAS-scores observed during the electrical pinprick-like stimulation, which showed no similar pattern to the EPs. Equally, the decrease in VAS-score directly after HFS, which might reflect a DNIC-like effect, was also not reflected in the evoked potential waveforms. Clearly the elicited brain response can be dissociated from the perceived pain intensity, something that has been observed more by a number of other authors, see for this topic [30]. In conclusion, we suggest that for studying heterotopic facilitation the evoked brain response is suitable and relevant for investigating plasticity at the level of the brain and is perhaps a more sensitive and reliable marker than the perceived pain intensity (e.g. VAS).

## Competing interests

The authors declare that they have no competing interests.

## Authors' contributions

EvdB was the initiator together with OWS for this study and developed the design. EvdB wrote the manuscript. CvH was involved in the development and discussion of the design and practically performed the study (data acquisition). CvH also contributed to the correction of the manuscript. CvR performed together with EvdB the data-analysis and helped with the interpretation and correction of the manuscript. OWS contributed to the development of the design, corrected all versions of the manuscript and gave final approval of the version to be published. All authors have read and approved the final manuscript.
